# Light-chain split luciferase assay implicates pathological NOTCH3 thiol reactivity in inherited cerebral small vessel disease

**DOI:** 10.1016/j.jbc.2025.108224

**Published:** 2025-01-24

**Authors:** Naw May Pearl Cartee, Soo Jung Lee, Emily Wu, Richard Sukpraphrute, Catherine Sukpraphrute, Jolie Greenbaum, Michael M. Wang

**Affiliations:** 1 Department of Neurology, University of Michigan, Ann Arbor, Michigan, USA; 2 Neurology Service, VA Ann Arbor Healthcare System, Department of Veterans Affairs, Ann Arbor, Michigan, USA; 3 Departments of Molecular and Integrative Physiology, University of Michigan, Ann Arbor, Michigan, USA

**Keywords:** CADASIL, NOTCH3, split luciferase, conformational change, cysteines, disulfide bonds

## Abstract

Stereotyped mutations in *NOTCH3* drive CADASIL, the leading inherited cause of stroke and vascular dementia. The vast majority of these mutations result in alterations in the number of cysteines in the gene product. However, non-cysteine-altering pathogenic mutations have also been identified, making it challenging to discriminate pathogenic from benign NOTCH3 sequence variants. Here, we present a method for quantitative assessment of NOTCH3 mutants, the light chain split luciferase (LSL) assay. In LSL, *NOTCH3* mutant fragments, cloned between a split luciferase open reading frame, are transfected into cells, producing secreted luciferase activity that is dependent on the normal structure of NOTCH3. Insertion of point mutants that cause CADASIL results in significantly lower activity. Using a panel of 47 sequences, we determined the sensitivity and specificity of LSL for pathogenic *NOTCH3* mutation discrimination to be 100% and 93%. LSL was also modestly successful in differentiating pathogenic proteins responsible for Marfan’s disease and Stiff Skin Syndrome. Two additional parameters from the LSL analysis (TCEP rescue of activity and secretion index) were also shown to be useful in characterizing NOTCH3 mutants. We show that the spacing and primary sequence of the light chain module is an important component of the LSL assay, as a single light chain cysteine is critical for pathogenic sequence discrimination. Furthermore, we show that the activity of CADASIL mutant reporters is amplified by the application of cysteine-reactive iodoacetamide, suggesting that LSL may be deployed to screen for novel compounds that suppress pathogenic conformations of NOTCH3.

CADASIL, the most common inherited cause of stroke and vascular dementia, results from a set of mutations in *NOTCH3* (1, 2). Molecular genetic analysis has suggested that the overwhelming majority of CADASIL mutations alter the number of cysteines of the gene product. The resulting mutant gene products contain an odd number of cysteine due to either loss or gain of a cysteine residue (3, 4). Notch proteins are highly conserved and composed of an array of EGF repeats, each consisting of three pairs of cysteines that form disulfide bonds (5). The changes in cysteine number in NOTCH3 linked to CADASIL has led to the hypothesis that mispairing of cysteine residues plays a key role in pathogenesis (6).

We recently described a detailed analysis of the effects of CADASIL mutations on protein mobility of the first three EGF repeats of NOTCH3 (7). All CADASIL mutants tested showed retarded mobility in native gels under non-reducing conditions, indicating that gross structural alterations result from disease mutations. A comprehensive analysis demonstrated that 18 of 18 loss of cysteine mutants generated structural alterations; meanwhile, the vast majority of non-cysteine mutant residues did not affect gel mobility. The residues to which lost cysteines were mutated did not affect the degree of mobility shift.

Furthermore, gel mobility assays enabled the evaluation of potential suppressor residues (*i.e.* second cysteine mutations paired with a disease allele) that reverse the gel mobility effects of single cysteine mutations (7). Notably, cysteine partners of the mutant cysteine residues were the most potent suppressors of mobility shifting. These studies validated the primacy of cysteine disulfides in maintaining the gross structural integrity of the NOTCH3 protein and indicated that mis paired cysteines may participate in pathological structural alterations. Since gel mobility shifting of NOTCH3 can be performed in most basic labs and identifies pathogenic mutants with high sensitivity and specificity, it is predicted to be an accessible and specific method to query the effects of individual NOTCH3 variants.

In the current investigation, we developed an alternate assay to gel mobility shift experiments to assess the effects of variants in the NOTCH3 protein. The new approach was motivated by the desire to (1) expand analysis beyond the first three EGF-like repeats that were the focus of gel shift assays; (2) develop methods to quantify the impact of disulfide bonding on NOTCH3 proteinopathy; and (3) permit scale-up of an assay that could be used in high-throughput screening.

Accordingly, we report a second accessible method of analyzing NOTCH3 protein variants that permits the quantification of pathological properties in NOTCH3: Light-chain Split Luciferase (LSL). LSL is composed of a custom vector for expressing variant gene fragments and a measurement workflow; these components form a flexible experimental platform for variant gene product analysis. We verified the applicability of LSL to differentiate CADASIL NOTCH3 mutants from wildtype and then applied LSL to NOTCH3 variants across several EGF domains to quantify their pathological potential. The novel LSL workflow generates three parameters that distinguish the impact of NOTCH3 variants on disulfide bonding and protein secretion. Furthermore, we report the adaptation of LSL to variants of FBN1 that are linked to independent diseases and discuss potential uses of LSL for high-throughput screening.

## Results

### Light chain split luciferase assay (LSL) vector design

**Figure 1:**
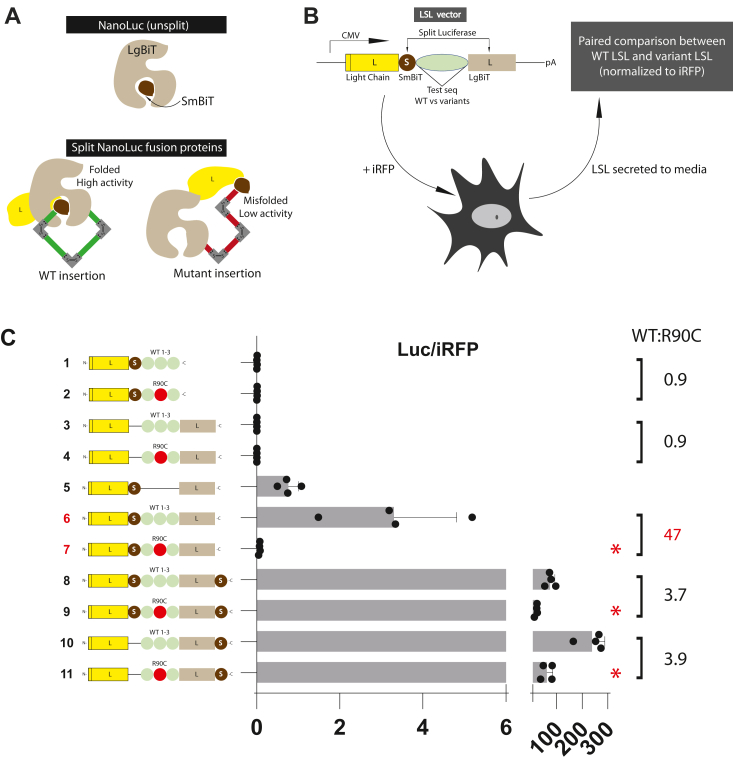
**Light chain Split Luciferase (LSL) system to discriminate between wildtype and mutant NOTCH3 protein sequences.***A*, representations of protein components are shown, with high activity wildtype nano luciferase represented at the *top*. In comparison, the split luciferase of LSL requires permissive conformations of the inserted test sequence that allows positioning of SmBiT in LgBiT to generate high activity (*green* insert of wildtype polypeptide is shown on the *left*). In contrast, if a variant causes large structural changes (*red insert* of pathogenic variants on the *right*), SmBiT is not able to interact with LgBiT to produce high activity. *B*, the design of the system includes a vector encoding components light chain variable domains of rabbit IgG (*yellow*), nano luciferase component SmBiT (*mocha*), test sequences of NOTCH3 or other genes (*green*), nano luciferase component LgBiT (*latte*). The LSL vector also includes a CMV promoter and polyadenylation signal sequence for expression by transfection into cells. A plasmid encoding iRFP is included for normalization of transfection efficiency. The effects of sequence variants cloned into the LSL vector are determined by referencing to wildtype LSL. *C*, comparison of LSL-NOTCH3 activity for wildtype and R90C canonical CADASIL mutation. Secreted nano luciferase activity from transfectants were compared after normalization to iRFP expression. The LSL-NOTCH3(1-3) wildtype construct generated high activity relative to the R90C mutant and to deletions of SmBiT and LgBiT. Activity generated by constructs containing wildtype NOTCH3 in LSL in which LgBiT was fused to SmBiT is also shown for comparison (bottom four). On the right, we quantify the ratio of iRFP normalized luciferase between WT and R90C mutant constructs. Data points are independent biological replicates with standard deviations displayed. We determined normality using the Shapiro-Wilk test. For two group comparisons, we used either the *t* test (unpaired; two-tailed) or the Mann-Whitney U test, as appropriate. Significant differences are indicated (∗) with *p* < 0.05.

Our overall objective was to generate a quantification tool to differentiate benign from pathogenic NOTCH3 protein. EGF-like domains, the site of all mutations that cause CADASIL, are conserved modules that each contain six cysteines that form three disulfide bonds (5, 8). The bonds are predicted to scaffold the peptide chain into a compact structure. A vast majority of pathogenic mutations in *NOTCH3* disrupt cysteine number (3, 9), which is predicted to distort disulfide bonding and to disrupt EGF-like domain structure. We reasoned that a split luciferase readout, incorporating NOTCH3 between luciferase sequences, could differentiate wildtype NOTCH3 protein sequences and mutant NOTCH3 sequences by virtue of differences in protein structure imparted by sequence variants (Fig. 1*A*).

The new split luciferase assay for investigation of NOTCH3 variants includes a genetic fusion of (1) light chain variable domain (L) of a rabbit antibody, (2) the SmBiT domain (S) of split nanoluciferase (NanoLuc) (10); (3) *NOTCH3* sequence variants; and (4) the LgBiT domain (L) of NanoLuc (10) (Fig. 1*B*; left side). Initial characterization of the LSL system was performed on NOTCH3 EGF domains 1 to 3, the site of both pathological and benign mutations in human NOTCH3. The system for evaluating NOTCH3 will be referred to as LSL-NOTCH3, and constructs featuring the insertion of coding sequences NOTCH3 EGF domains 1 to 3 are referred to as LSL-NOTCH3(1-3). After transfection of LSL-NOTCH3(1-3), secreted protein activity can be quantified for luciferase activity. The efficiency of transfection is normalized by co-transfection with iRFP (11, 12), which can be quantified by plate scanning without cell destruction (Fig. 1*B*).

The light chain antibody fragment was originally conceived to promote the secretion of the recombinant protein to the media and to provide a target for commonly available antibodies that enable quantification of recombinant protein. The choice of the split NanoLuc was motivated by its high level of activity (13, 14) and prior work showing that spatial approximation of SmBiT and LgBiT sequences reconstitutes activity (10). Both NanoLuc polypeptides are devoid of cysteines and are predicted to be insensitive to reducing agents. We cyclically permuted the two sequences so that SmBiT is produced an N-terminal to LgBiT, which has been effective in other split protein systems (15).

We indeed found that the genetic fusion LSL-NOTCH3(1-3) generates protein with high enzymatic activity detectable in secreted media (Fig. 1*C*; construct 6). No activity was generated above background when SmBiT or LgBiT was deleted (Fig. 1*C*; constructs 1–4), but the LSL-NOTCH3(1-3) activity was lower than that generated by the fusion of NOTCH3 sequences to intact nanoLuc (Fig. 1*C*; constructs 8–11). These studies are consistent with wildtype NOTCH3 EGF domains 1 to 3 folding in a manner that would permit spatial alignment of SmBiT and LgBiT, reflected by reconstitution of luciferase activity.

### Light chain split luciferase assay (LSL) for quantification of abnormalities in NOTCH3

To test the effects of pathogenic mutations on LSL-NOTCH3 activity, we compared the activity generated by a prototypical CADASIL-causing R90C gain of cysteine mutation (Fig. 1*C*). Mutant R90C LSL-NOTCH3(1-3) transfected cells produced markedly suppressed levels of luciferase activity (normalized to iRFP) that was consistent with the blocking of secreted LSL activity by conformational changes of the split luciferase module (Fig.. 1*C*; construct 7). High level activity was generated by mutant R90C sequences fused to intact nanoLuc, demonstrating that the suppressive action of the mutant largely depended on the split luciferase domain (Fig. 1*C*; constructs 9 and 11). There was a 47-fold decrease in LSL-NOTCH3(1-3) activity when the CADASIL mutant was compared to wildtype (Fig. 1*C*; right side).

**Figure 2:**
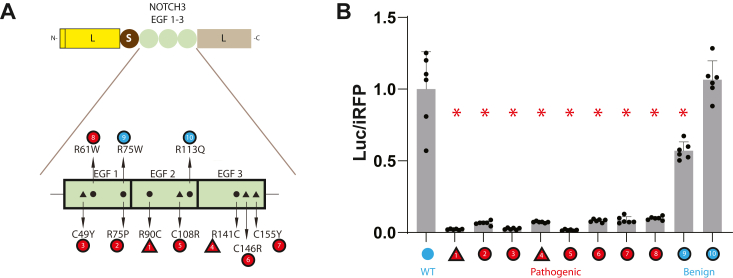
**Ability of LSL-NOTCH3 to discriminate between wildtype NOTCH3, CADASIL mutants, and non-pathogenic variants.***A*, schematic showing LSL-NOTCH3(1-3) vector used to analyze NOTCH3 EGF 1-3 variants. Cysteine (*triangles*) and non-cysteine (*circles*) residues were involved in pathogenic mutations. Amino acid changes are shown with pathogenic changes in *red* and non-pathogenic variants in *cyan*. *B*, activity in media (iRFP normalized) of LSL-NOTCH3(1-3) transfected cells are shown, corresponding to variants in (*A*). All iRFP controlled values were referenced to WT (set at 1.0). Wildtype, pathogenic, and benign mutations are shown. Data points are independent biological replicates with standard deviations displayed. We determined normality using the Shapiro-Wilk test. Significant differences were determined for parametric data using one-way ANOVA with the Dunn’s multiple comparisons test. Non-parametric data were evaluated using the Kruskal-Wallis test. All pathogenic mutans showed significant differences from wildtype as indicated (∗) with *p* < 0.05. Benign variant R113Q was not significantly different from wildtype.

To test whether the LSL-NOTCH3 system was capable of broadly differentiating pathogenically altered protein from wildtype and benign variants of NOTCH3, we generated a series of pathogenic (cysteine altering and non-cysteine altering [R61W and R75P]) and benign sequence variants that were transfected into cells (Fig. 2*A*). The conditioned media were tested for secreted luciferase activity (normalized to transfected iRFP levels), which was compared to wild-type activity. As shown in Figure 2*B* (showing wildtype value set to 1), all pathogenic LSL-NOTCH3 variants generated significantly decreased levels of luciferase activity. In contrast, benign variants generated levels that were similar to wild type.

We concluded that secreted LSL-NOTCH3 activity is a parameter that readily discriminates between benign and pathogenic mutants (referred to as parameter 1). Two additional parameters generated by the LSL system will be described later.

### Effect of specific amino acid changes on LSL-NOTCH3 activity

**Figure 3:**
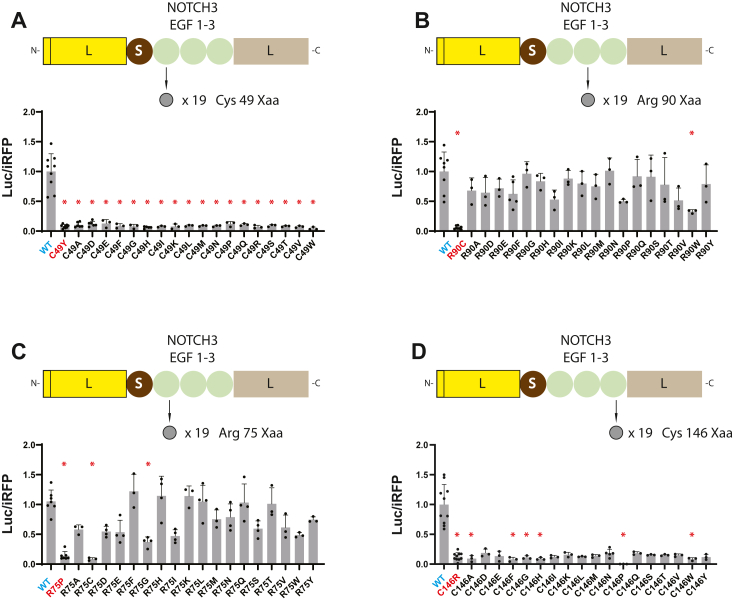
**Effect of mutations at key NOTCH3 residues on LSL-NOTCH3 activity.** Mutations were introduced into the NOTCH3 EGF 1-3 sequences of LSL-NOTCH3(1–3) in order to assess the effects of specific amino acid residues on LSL activity. Constructs are shown above each group of data which represents the iRFP normalized secreted luciferase activity for wildtype and mutants at NOTCH3 residues 49 (*A*), 90 (*B*), 75 (*C*), and 146 (*D*). The wildtype residues are C49, R90, R75, and C146, which were compared to mutations to all 19 other amino acids. Established pathological mutants are shown in *red* and WT is in *cyan*. Data points are independent biological replicates with standard deviations displayed. We determined normality using the Shapiro-Wilk test. Significant differences were determined for parametric data using one-way ANOVA with the Dunn’s multiple comparisons test. Non-parametric data were evaluated using the Kruskal-Wallis test. Significant differences from wildtype are indicated (∗) with *p* < 0.05.

**Figure 4:**
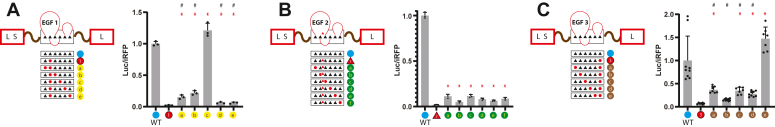
**Mapping of second cysteine mutations that suppress pathological NOTCH3 variant LSL-NOTCH3 function**. Three mutations in NOTCH3 EGF 1-3 were analyzed and displayed in separate panels. Each panel shows, on the *left*, a schematic of mutations that were introduced into LSL-NOTCH3(1-3) and denotes the EGF repeat that harbors the mutation, depicted by a *red* circle (loss of cysteine) or by a *red* triangle (gain of cysteine). *Black* triangles represent the six cysteines of wildtype NOTCH3 in each repeat. The *top* construct (*cyan dot*) is the wildtype reference, the second construct is the established CADASIL mutant (in *red*), and all other constructs are second mutations in each of the remaining cysteine positions (changed to serine). Corresponding secreted LSL activity generated by constructs, normalized to iRFP expression, was determined for each construct, and all values were referenced to the WT construct (value of 1.0) shown in the chart to the right in each panel. The CADASIL mutants analyzed included C49Y (*A*; EGF 1), R90C (*B*; EGF 2), and C155Y (*C*; EGF 3). Double mutants with significant differences from established CADASIL mutants are indicated (partial rescue of activity, ∗). Double mutants with significant differences from the ideal double mutant (*yellow* c and *brown* e double mutants in (*A* and *C*)) are indicated (weaker than ideal suppressor, #). Data points are independent biological replicates with standard deviations displayed. We determined normality using the Shapiro-Wilk test. Significant differences were determined for parametric data using one-way ANOVA with the Dunn’s multiple comparisons test. Non-parametric data were evaluated using the Kruskal-Wallis test. Significance was considered as *p* < 0.05.

Previous gel shift studies highlighted the importance of cysteines in mutant NOTCH3 protein structure (7). To test the concordance between LSL and structural (gel shift) assays, we generated LSL constructs that corresponded with gel shift experiments from (7) to test 1) the effect of different residues in positions 49, 75, 90, and 146 (Fig. 3); and 2) the potential suppressor effects of second cysteine mutations within EGF repeats (Fig. 4).

Figure 3 shows the results of a single mutant analysis of LSL-NOTCH3(1-3) activity. We analyzed the effects of mutations at residues 49 and 146, which are cysteine in the wildtype protein and are the sites of CADASIL mutations C49Y and C146R. All non-cysteine replacements at residue 49 yielded decreased secreted luciferase activity that was significantly changed from wildtype (Fig. 3*A*). All non-cysteine replacements at residue 146 resulted in a large drop in activity compared to wildtype, with several, but not all, of these significant (Fig. 3*D*).

Although most CADASIL mutations cause a gain or loss of cysteine, there are notable exceptions which include the R75P mutation that has been described in Asian populations. In prior work, we showed that NOTCH3 migrates abnormally in gels when position 75 is mutated to proline, cysteine, or glycine, indicating that selective changes trigger protein structural alterations that may relate to pathology. We introduced all twenty amino acids into position 75 in LSL-NOTCH3(1-3) and assessed secreted luciferase activity (Fig. 3*C*). Only proline, cysteine, and glycine mutants induced reductions in activity, confirming the selective nature of changes in this position that result in structural alterations of NOTCH3.

In Figures 1 and 2, we showed that a pathological gain of a cysteine at position 90, corresponding to the canonical R90C CADASIL mutation, resulted in marked suppression of secreted luciferase activity. In prior work, we found that gel shift abnormalities resulted only from mutations to cysteine at this location. In the LSL-NOTCH3(1-3) experiment where position 90 was mutated to all possible amino acids (Fig. 3*B*), we found significantly lowered activity with mutations to cysteine and tryptophan; all other residues resulted in activities that were not significantly different from wildtype. This largely matches gel shifting data and again emphasizes the importance of cysteine changes in driving NOTCH3 structural alterations.

Figure 4 shows experiments in which we used LSL to potentially confirm and extend prior work that led to the discovery of suppressor mutations that reverse CADASIL abnormalities. Cysteine mutations may drive CADASIL pathology by inducing a cysteine imbalance in which free thiol groups are free to react with cysteines within NOTCH3 or within other proteins *via* new, dysfunctional disulfide bonds (6). If that is the case, CADASIL mutant proteins that harbor second mutations of specific cysteines are predicted to suppress dysfunctional conformations. In our previous study (7), we indeed identified EGF repeat-specific second mutations in cysteine residues that reversed gel shifts caused by CADASIL mutations C49Y, R90C, and C155Y. We cloned these mutants and all potential suppressors cysteine mutations into LSL-NOTCH3(1-3) to quantify the impact of double mutations (relative to single CADASIL mutations). As shown in Figure 4*A*, in the context of the C49Y mutant (which results in the loss of the second cysteine of EGF1), the most effective second mutations that reversed suppressed LSL activity a cysteine to serine alteration in the fourth cysteine residue of EGF1. This residue is normally paired with the second cysteine. Other loss of cysteine second mutations also resulted in significant increases in suppressed luciferase activity but suppressed the mutant defect to a much lower degree. For the gain of mutation R90C mutant (Fig. 4*B*), all second cysteine mutants modestly suppressed mutation-related effects on the LSL reporter, with none exhibiting significant superiority over others. For the loss of cysteine mutant C155Y, which causes a loss of the sixth cysteine of EGF3, we found that all cysteine mutations in EGF3 counteracted the loss of LSL activity caused by the CADASIL mutations (Fig. 4*C*). However, the mutation in the fifth cysteine, which normally pairs with the C155 residue, was the only one that completely restored reporter activity. Overall, results from the LSL assay demonstrate that mutation of the cognate cysteine that is altered in CADASIL had a marked effect on the activity of the reporter, which correlates with structural investigations using gel shifts that support the same model. In addition to identifying the most potent suppressor mutations, the use of LSL appears to support that other residues have partial suppressor effects and suggests that the assay may be more sensitive than gel shift assays.

### Role of disulfides in LSL inhibition by mutant NOTCH3

**Figure 5:**
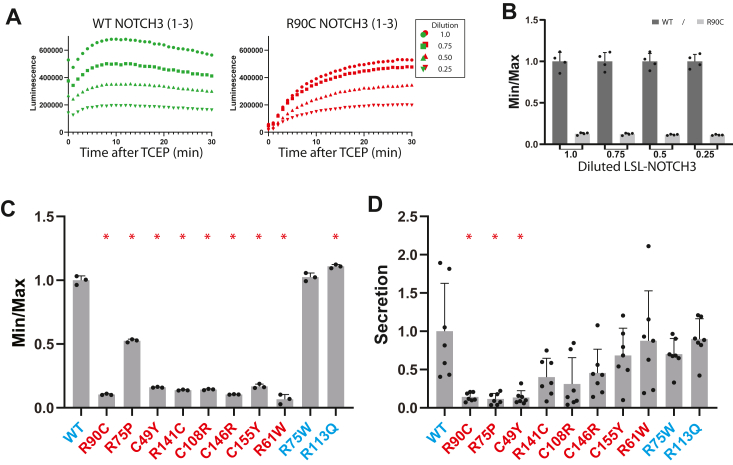
**Effect of pathogenic *versus* benign NOTCH3 variants on additional parameters of LSL-NOTCH3 activity: Min/Max and Secretion Index.***A*, TCEP-responsiveness of LSL-NOTCH3 activity generated by wildtype NOTCH3 EGF 1-3 *versus* the CADASIL mutant R90C was determined by addition of 2 μl TCEP (31.25 mM) to the nano luciferase assay mixture. The amount of enzyme from the same assay was reduced in increments of 25%, and time courses of activity were displayed in the same graph. Each series of activities represents undiluted conditioned media, 75% media, 50% media and 25% media (in descending order of activity, with WT (*green*) and R90C mutant (*red*). *B*, the minimum activity (Min; time 0 prior to TCEP addition) and the maximum activity (Max) after TCEP addition was used to calculate the Min/Max parameter. The paired Min/Max values are displayed for both the wildtype LSL-NOTCH3(1-3) WT *versus* R90C CADASIL mutant for all dilutions shown in (*A*). LSL activities produced by different concentrations of media did not affect the Min/Max parameter. *C*, the Min/Max (parameter 2) of LSL-NOTCH3(1-3) for an expanded set of NOTCH3 mutants in EGF 1 to 3 were assessed by challenging media from transfectants with TCEP. The same mutants as in Figure 2 were used, representing both pathological (*red*) and benign (*cyan*) NOTCH3 variants. The Min/Max values were all referenced to WT Min/Max. *D*, the Secretion Index (parameter 3) for all mutants analyzed in (*C*) was determined by comparison of the maximum TCEP-induced activity secreted into the media to the maximum TCEP-induced activity produced by cell lysates. Data points are independent biological replicates with standard deviations displayed. We determined normality using the Shapiro-Wilk test. Significant differences were determined for parametric data using one-way ANOVA with the Dunn’s multiple comparisons test. Non-parametric data were evaluated using the Kruskal-Wallis test. For analyses of (*C* and *D*), significant differences from wildtype are indicated (∗) with *p* < 0.05.

Gel mobility shifting abnormalities of mutant NOTCH3 are eliminated by reducing agents, an indication that abnormal disulfide bonds of mutant NOTCH3 participate in protein conformational changes (7). We expected that if NOTCH3 disulfide status was related to suppression of luciferase activity, treatment with the reducing agent TCEP would rescue activity. To test how secreted LSL-NOTCH3 proteins respond to reducing agents, conditioned media including LSL proteins was challenged with TCEP, and activity was followed over time (Fig. 5*A*). The wild-type LSL-NOTCH3(1-3) modestly increased in activity (∼30% increase), while the R90C LSL-NOTCH3(1-3) sharply increased activity (∼1000% increase); increases were seen at all concentrations of protein tested (multiple curves in Fig. 5*A*). When displayed as the ratio of the minimum activity to the plateau activity (Min/Max), the TCEP-inducible LSL levels showed much greater levels for mutant protein at all enzyme dilutions. The TCEP-inducible LSL levels, corresponding to low Min/Max values, were independent of protein concentration for both wildtype and R90C protein (Fig. 5*B*). Conceptually, we consider the activity after TCEP to be the total potential activity, whereas the activity before TCEP is considered an activity that is actively blocked by abnormal disulfide bonding involving mutant NOTCH3.

To determine if elevated TCEP-inducibility of LSL-NOTCH3 luciferase activity is a feature of other pathogenic mutants of NOTCH3 and if it is specific for pathogenic vs benign variants, we expanded the analysis of LSL-NOTCH3 variants in Figure 5*C*. After transfection of LSL-NOTCH3 constructs, we determined the Min/Max levels of activity after TCEP treatments. All experiments included corresponding wildtype constructs as a reference, which was expected to generate the highest Min/Max values (least TCEP-inducible). For EGF-like repeats 1 to 3 of NOTCH3, we observed high Min/Max values for wildtype and benign NOTCH3 variants. The Min/Max values were consistently suppressed for all pathological mutants, indicating that their activities were increased by the reduction of the reporter proteins. Overall, there was excellent concordance between pathogenicity of mutations and TCEP-inducibility, indicating that the Min/Max value is a second parameter that reflects disease-related changes in NOTCH3 structure (referred to as parameter 2).

### Protein secretion abnormalities in subsets of mutant NOTCH3

We noted that for the pathological mutant R75P, the overall Min/Max levels with TCEP challenge were only moderately reduced compared to wildtype, indicating that some pathological mutants generate a surprisingly high fraction of properly folded protein. Because all pathological mutants generated lower total secreted LSL activity, we hypothesized that the low secreted level of LSL activity for these mutants may result from poor secretion. To test this, we measured the ratio of LSL in the media to that remaining in cells.

Transfections with wild-type and mutant LSL-NOTCH3 were analyzed by harvesting media and cells. Media was isolated from the mixture by centrifugation, and the cell pellet was separately analyzed by lysis in luciferase assay buffer. All luciferase values were obtained after treatment with TCEP to capture the total potential luciferase activity, irrespective of disulfide-dependent conformational pathology. In all experiments, the ratio of media to cell-associated activity was compared to that of the corresponding wildtype LSL-NOTCH3 (Fig. 5*D*).

For benign variant EGF-like repeat clusters, secretion ratios were the same as that of wild type. However, some pathogenic mutations resulted in a decreased secretion ratio (R90C, R75P, and C49Y). However, not all pathogenic mutations resulted in adecreased secretion. Based on these data, the secretion ratio of NOTCH3 variants in the LSL system serves as a specific parameter for pathogenicity. The sensitivity of this third LSL parameter (referred to as parameter 3) was lower than that of the first two parameters in this limited mutation set.

In total, the LSL-NOTCH3 system generated three parameters that are useful for the discrimination of pathogenic and benign NOTCH3 variants: (a) secreted LSL activity; (b) Min/Max value (TCEP-inducible activity); and (c) secretion ratio. In the Discussion, we describe the significance and utility of these three parameters.

### Importance of the light chain module of LSL in pathological discrimination

Since there are no cysteines in SmBiT and LgBiT, the alteration of activity of LSL-NOTCH3 in the presence of TCEP is dependent on disulfide bonds in NOTCH3 and/or in the antibody light chain. To further characterize the basis of NOTCH3 variant-mediated regulation of LSL activity, we generated a series alteration in the light chain module of LSL and tested their impact on the magnitude of discrimination between wildtype and R90C NOTCH3 sequences.

**Figure 6:**
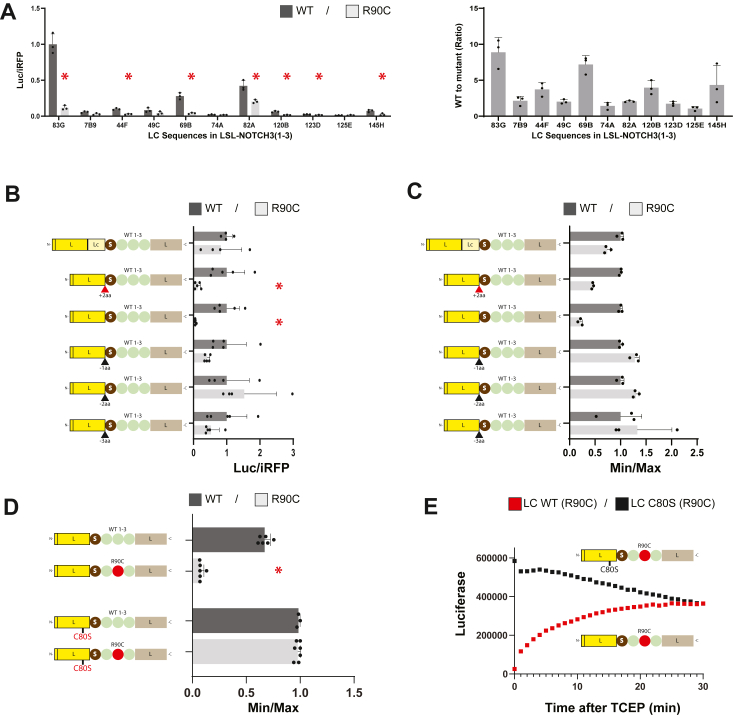
**Mapping locations within LSL important for pathogenic *versus* benign discriminatory capacity.** Sequences in the light chain portion of LSL were altered to determine sequences that were important for high discriminatory function of LSL-NOTCH3. *A*, Figure 1, Figure 2, Figure 3, Figure 4, Figure 5 used the 83G light chain variable sequences in LSL-NOTCH3(1-3) constructs. Here, the light chain coding sequence was replaced by a series of rabbit monoclonal antibody light chain variable sequences listed on the x-axis. Secreted activity was normalized to iRFP for both WT and R90C versions of each light chain construct are displayed in the chart on the *left* which shows all constructs yield higher values for WT over mutant NOTCH3. On the *right* chart, the ratio of WT to mutant generated luciferase values is shown to demonstrate the magnitude of discrimination for each light chain. *B*, constructs using 83G light chain in the LSL-NOTCH3 backbone were generated with small insertions or deletions 5′ to the SmBiT sequence. WT or R90C NOTCH3 EGF 1-3 reporter activities were determined and compared in the chart, which shows each value referenced to the respective WT construct (value set to 1.0). *C*, the effect of TCEP-mediated reduction on activities from (*B*) were determined by adding TCEP to the reaction mixture and determining the Min/Max value. The Min/Max values were referenced to WT (set to 1.0). Constructs with the largest differences between WT and R90C were considered the best at discrimination between WT and pathological mutants. *D*, to assess the role of the unpaired cysteine residue at position 80 of rabbit light chain, constructs were generated in LSL-NOTCH3(1-3) with mutation C80S. Analysis of the Min/Max parameter of these constructs that incorporated WT and NOTCH3 R90C was performed by measurement of luciferase activity over time after addition of TCEP. The time-dependent activity after addition of TCEP for a representative set of R90C mutants with or without C80S is shown in (*E*). Data points are independent biological replicates with standard deviations displayed. We determined normality using the Shapiro-Wilk test. For two group comparisons, we used either the *t* test or the Mann-Whitney U test, as appropriate. Significance (∗) was considered as *p* < 0.05.

First, we substituted the light chain variable sequence of LSL. The original clone used the rabbit variable sequence from 83G, a monoclonal antibody generated against NOTCH3. Other variable sequences derived from the same animal after immunization were tested with wildtype and R90C NOTCH3 sequences. Compared to constructs with other light chain sequences, 83G generated the greatest luciferase activity in secreted media (left panel, Fig. 6*A*). Furthermore, 83G-derived LSL clones produced the magnitude of discrimination between wildtype and R90C sequences (ratio of wildtype to R90C shown in the right panel of Fig. 6*A*). As such, among a series of light chain variants, 83G appeared to exhibit the best characteristics for studying NOTCH3 among those testes.

Second, we replaced the light chain variable portion of LSL with the complete light chain (variable plus constant domains [LC]; blue bar of Fig 6*B*). In the presence of a complete light chain, there was no difference in secreted protein activity between wildtype and mutant NOTCH3 fusions. Moreover, the activation of pathological mutant LSL-NOTCH3 in this context by TCEP was no longer present (blue bar of Fig. 6*C*).

Third, we made small insertions and deletions N-terminal to SmBiT in the LSL construct (green and black bars of Fig. 6, *B* and *C*). The addition of two amino acids to the light chain of the LSL reporter reduced the discriminatory capacity of the system (green bar of Fig. 6, *B* and *C*). The mutant-dependent reporter expression levels (Fig. 6*B*) and TCEP responsiveness (Fig. 6*C*) of the mutants were no longer present with deletions of 1 to 3 amino acids of the light chain module. Overall, these findings indicated that the light chain variable domain spacing relative to NOTCH3 may influence the discriminatory capabilities of the system.

A unique feature of rabbit IgG light chains is the presence of a disulfide bond that bridges the variable and constant domains; this cysteine bridge is absent from human IgG. In LSL, the absence of the constant domain removes the bonding partner for a conserved cysteine at residue 80. We considered the possibility that C80 (numbering of amino acids without secretion signal peptide) of LSL could participate in disulfide bonding with pathological mutant NOTCH3, which is known to harbor free thiols and likely engages in abnormal disulfide bridges (6, 7). The formation of C80 to mutant NOTCH3 disulfides could dramatically strain the SmBiT domain of the reporter protein and strongly inhibit split luciferase activity; the suppression of activity should be relieved in the presence of TCEP. Indeed, when we introduced C80S mutations into LSL, the mutant NOTCH3 reporter did not respond to TCEP (Fig. 6, *D* and *E*). In sum, we show that (1) spacing of NOTCH3 in relation to the light chain and (2) a single residue (C80) in LSL that may form a disulfide bridge with mutant NOTCH3 are key factors that make the LSL system useful for NOTCH3 sequence evaluation.

### Application of LSL to other sets of NOTCH3 EGF repeats

**Figure 7:**
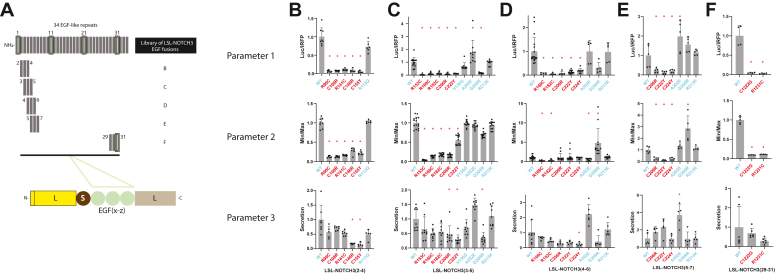
**LSL-NOTCH3 activity with insertion of additional regions of NOTCH3.***A*, The schematic of NOTCH3 and its 34 EGF repeats is shown above representations of subdomains of NOTCH3 selected for additional LSL-NOTCH3 analysis. Each subdomain is composed of three adjacent EGF repeats and included analysis of EGF (29–31) in the C-terminal region of NOTCH3. Domains were cloned in the LSL vector as before. Activity of the reporters were compared for WT, pathological mutants (*red*) and benign variants (*cyan*) by transfection and analysis of three parameter, iRFP normalized luciferase activity (parameter 1), TCEP-responsiveness (Min/Max; parameter 2), and Secretion Index (parameter 3). For all parameters in each chart, we referenced valued to WT. EGF clusters analyzed included 2 to 4 (*B*), 3 to 5 (*C*), 4 to 6 (*D*), 5 to 7 (*E*), and 29 to 31 (*F*). Data points are independent biological replicates with standard deviations displayed. We determined normality using the Shapiro-Wilk test. Significant differences were determined for parametric data using one-way ANOVA with the Dunn’s multiple comparisons test. Non-parametric data were evaluated using the Kruskal-Wallis test. For (B-F), significant differences from wildtype are indicated (∗) with *p* < 0.05.

The EGF repeats of NOTCH3 have conserved features but are heterogeneous, with differences in pathogenicity of mutations in NOTCH3, which depends on the location within the protein sequence (16, 17, 18). To determine potential differences in sequence characteristics, we examined the basal features of WT NOTCH3 EGF triplets across the entire ectodomain for any of the three LSL parameters. The ectodomain was segmented into clusters of three EGF-repeats, and constructs were expressed in cells (Fig. 7*A*). Secreted luciferase activity (normalized to iRFP activity) was determined for wildtype and mutant NOTCH3 ectodomain fragments (Fig. 7*B*). The Min/Max values and the secretion parameters were also determined across domains for different mutants, relative to wildtype.

Without exception, decreased secreted luciferase was noted for constructs that included pathogenic mutations in EGF-like repeats 2 to 4, 3 to 5, 4 to 6, 5 to 7, and 29 to 31; with benign variants, there was only a single construct that generated a decrease in activity (G209R in EGF3-5; Fig. 7*C*). Decreases in Min/Max and secretion parameters were noted in pathogenic mutants but was less sensitive to the presence of pathogenic mutations. We aggregated all of the constructs together and determined that the overall sensitivity and specificity in discriminating pathogenic from non-pathogenic NOTCH3 mutations were 100% and 93%.

### Application of LSL to other disorders

Other degenerative disorders are caused by mutations in EGF-repeats encoded by other genes, including Marfan disease and Stiff Skin Syndrome (SSS), which are caused by mutations in *FBN1*. To determine whether LSL could be applied to these disorders, we cloned a sample of mutations in *FBN1* into the LSL framework (LSL-FBN1) and compared activity parameters generated from disease-causing mutants to those of the corresponding wildtype sequence.

**Figure 8:**
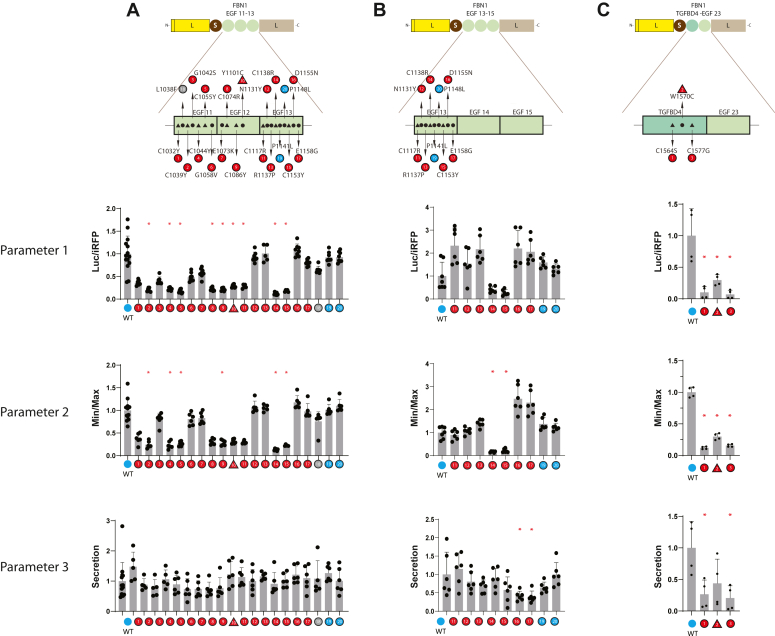
**LSL-FBN1 activity corresponding to variants associated with Marfan’s disease and stiff skin syndrome.** EGF domains of FBN1 were inserted into the LSL vector and transfected into cells. Mutations that are considered pathogenic and benign variants were compared to wildtype sequences. Secreted activity was analyzed, along with min/max and secretion indices. EGF domains 11 to 13 and 13 to 15 of FBN1 were used to examine variants linked to Marfan’s disease (*A* and *B*), and TGFBP domain 4 with EGF domain 23 (residues 1528–1651) were used to test activity related to mutations causing stiff skin syndrome (*C*). Data points are independent biological replicates with standard deviations displayed. We determined normality using the Shapiro-Wilk test. Significant differences were determined for parametric data using one-way ANOVA with the Dunn’s multiple comparisons test. Non-parametric data were evaluated using the Kruskal-Wallis test. Significant differences from wildtype are indicated (∗) with *p* < 0.05.

Mutations in FBN1 EGF-repeats 11 to 13 and 13 to 15 were analyzed by LSL-FBN1 and three parameters of LSL analysis are shown (Fig. 8, *A* and *B*). This revealed that some, but not all, of the FBN1 pathogenic mutations correspond with alterations in iRFP-normalized luciferase activity. Similarly, some, but not all, of the pathogenic mutations yield differences in Min/Max (parameter 2) and none produced differences from wildtype in secretion ratios (parameter 3). All benign variants yielded three parameters that were not different from wild type. Mutations in *FBN1* that are linked to SSS (TFGBD4) showed alterations in all three parameters tested in an LSL-FBN1 reporter that included TGFBD4 and the adjacent EGF23 domain (Fig. 8*C*).

### Extrinsic regulators of mutant NOTCH3 structural transformation

**Figure 9:**
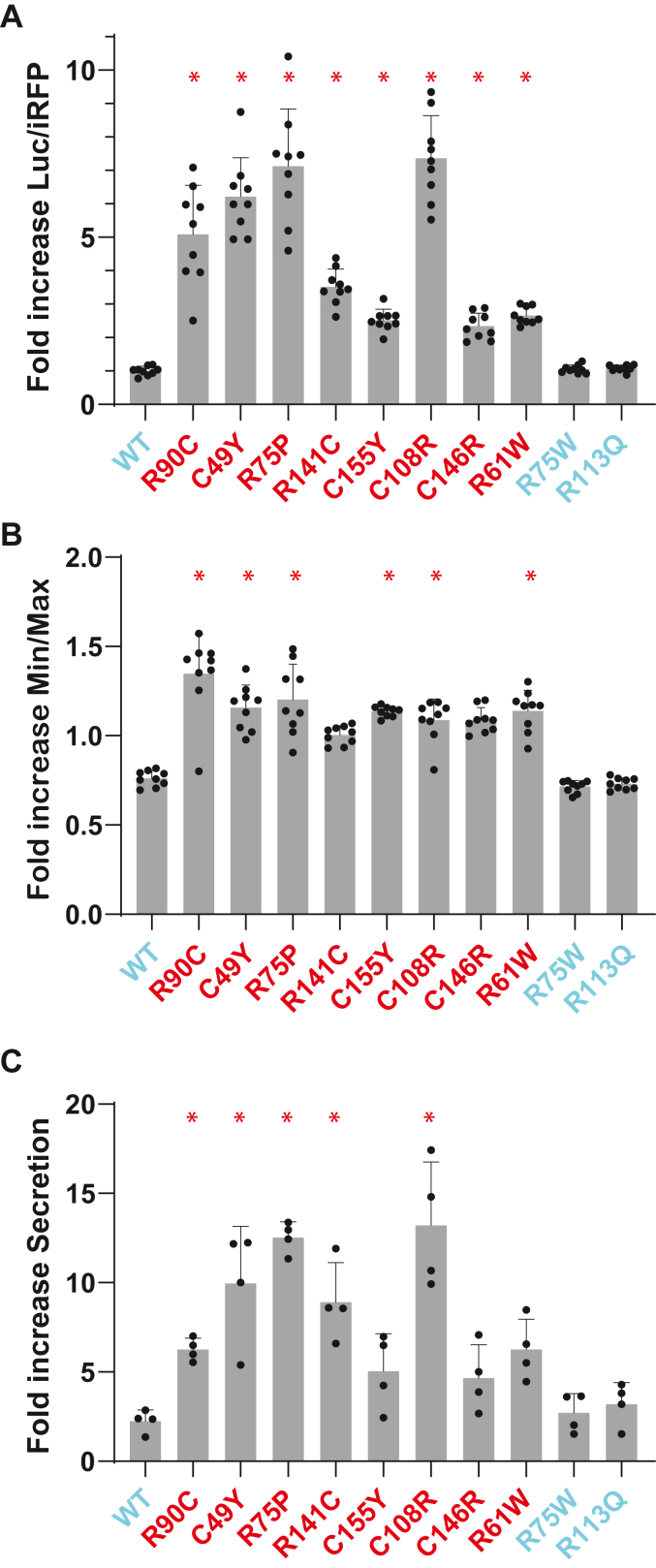
**Effect of iodoacetamide on LSL-NOTCH3 variant activity.** Cells were transfected with LSL-NOTCH3 EGF 1-3 constructs encoding wildtype, pathogenic CADASIL mutations (*red*), or benign variants (*cyan*). Cells were treated for 2 h by addition of iodoacetamide (10 μM) or water, and secreted luciferase activity determined. All values were referenced to water treatment and thus display the ratio of activity with to without iodoacetamide. *A*, as before, secreted luciferase values were normalized to iRFP to determine parameter one increases. *B*, to determine changes in parameter 2, Min/Max increases after addition of iodoacetamide are displayed. *C*, for parameter three assessment, the Secretion Index was determined for NOTCH3 variant reporters in cells treated with and without iodoacetamide. The ratio of treatment to water control is shown in the chart. At least three experiments were performed; data points are independent biological replicates with standard deviations displayed; we determined normality using the Shapiro-Wilk test. Significant differences were determined for parametric data using one-way ANOVA with the Dunn’s multiple comparisons test. Non-parametric data were evaluated using the Kruskal-Wallis test. Significant differences in iodoacetamide stimulated activity for variants *versus* WT control (∗) are indicated for *p* < 0.05.

To investigate potential modulators of mutant NOTCH3 structure in cells, we transfected LSL-NOTCH3(1-3) constructs into cells and then challenged cells with iodoacetamide, a general thiol alkylator. We reasoned that if free thiols of NOTCH3 underlie repression of pathological LSL reporter activity, capping thiols may increase luciferase activity. Further, this experiment could test the feasibility of the LSL system to screen for compounds that attenuate the impact of mutant NOTCH3. Figure 9 shows that treatment of cells with iodoacetamide (10 μM) for 2 h resulted luciferase activity increases that were greater for CADASIL mutants than wildtype NOTCH3 and benign variant LSL constructs. In addition, the Min/Max values (parameter 2) and secretion indices (parameter 3) were significantly increased for multiple mutants; there were no changes in these parameters for wildtype and benign LSL-NOTCH3 reporters. These studies suggest that the alkylation of cysteine thiols can rescue LSL activity of pathological NOTCH3 mutants.

## Discussion

Although the initial molecular genetics of inherited small vessel disease implicated cysteine residues of NOTCH3 as disease-initiating factors, altered biochemical features of mutant NOTCH3 protein have only recently emerged. This study provides a new approach to characterizing differences between wild type and pathogenic NOTCH3 features using a simple, quantifiable transfection assay. We demonstrate that LSL is capable of clearly differentiating benign from pathogenic changes in NOTCH3 using three parameters that result from luciferase measurements. The assay can be used to identify conditions that suppress NOTCH3 protein abnormalities and may be applied to other genetic disorders.

### LSL: a new assay for differentiating pathogenic polypeptide sequences

For NOTCH3 variants, LSL-NOTCH3 provides a high level of discriminatory capacity (wildtype and benign variants vs. pathogenic variants). There was also strong alignment between the investigation of NOTCH3 mutant sequences that do not correspond to variants found in the population and prior work using a gel shift assay to assess protein configurations. For example, single mutant changes at residues 49, 75, 90, and 146 showed a strong preference for cysteines in both this work and earlier gel shift studies. Specific, unexpectedly significant changes, such as R75G, demonstrated alterations in both protein conformation in gel shifts and by LSL. Suppressor studies in the LSL matched gel shift suppressor studies; both approaches indicate that the strongest suppressor mutants are found when both cysteines that are normally paired are mutated. In case studies, it appears that LSL may have increased sensitivity compared to gel shifting. Previous studies only showed significant suppression of gel shift abnormalities with mutation of paired cysteines (eg mutations of the second and fourth cysteine in C49Y proteins), but in LSL analyses, all three mutants examined in Figure 4 demonstrate broad second cysteine-mutation sensitivity, with paired cysteines showing numerically highest suppression. As such, LSL may be more compatible than gel shift assays for expanded efforts to identify suppressor mutations or small molecules that alter NOTCH3 pathogenic features.

Three parameters are obtained in each LSL assay: (1) the total activity of oxidized reporters (min/iRFP); (2) the fraction of reporters that is active under oxidized *versus* reduced conditions (Min/Max); and (3) the fraction of reporter that is secreted *versus* cell-associated (secretion index). One or more of these parameters is abnormal in each NOTCH3 mutant, but mutations alter different combinations of these parameters, indicating that each NOTCH3 mutant may have distinct properties.

**Figure 10:**
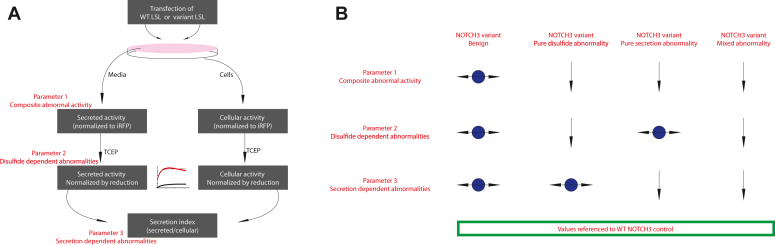
**Framework for interpretation of LSL assays for NOTCH3 variants.***A*, a suggested workflow for analyzing the effects of gene variants is shown. LSL reporters can be generated that correspond to genetic variants and compared by transfection, followed by analysis of luciferase activities after TCEP treatment of media and cell lysates. *B*, a framework to account for the results of LSL analysis of NOTCH3 benign and pathological mutants is shown. The cellular and biochemical effects of each variant result in differing patterns of parameter 1 to 3 alteration as discussed. It is proposed that pathological variants of NOTCH3 alter disulfide arrangements and/or protein secretion.

Our working model for what the assay is reading out is shown in Figure 10. We conjecture that the normalized activity of LSL-NOTCH3 (parameter 1) is the overall level of active LSL enzyme, which is modulated by (a) the fraction of LSL that achieves conformations that produce LSL activity and (b) the secretion efficiency of the protein. Meanwhile, the Min/Max ratios of LSL-NOTCH3 activity (parameter 2) represents the fraction of LSL that achieves conformations that produce LSL activity; the max levels after TCEP treatment, as shown above, likely represent amount of LSL that is in a flexible state, unhindered by disulfide bonding, while the min level is the level of LSL that is active when the NOTCH3 module is disulfide bonded. Mutagenesis of C80 of the light chain suggests that when a pathological mutant of NOTCH3 (with free thiols) is part of LSL-NOTCH3, it is capable of bonding with C80, constraining the LSL-NOTCH3 protein in a manner that prevents SmBiT from LgBiT interaction. Finally, the secretion index (parameter 3) is simply the amount of protein secreted *versus* withheld in cells.

Utilizing all three LSL parameters in the context of NOTCH3, we offer several conclusions. First, parameter one offers the most sensitive screen for pathogenicity of NOTCH3, since it integrates disulfide pathology and secretion pathology into a single assessment (sensitivity was 100% over all 5 EGF combinations). Second, pathological NOTCH3 mutants demonstrate different degrees of alteration of parameter two and parameter 3 (sensitivities of 93% and 29% over all 5 EGF combinations). The specificity of the three parameters for discrimination of pathological mutations were 93%, 96%, and 89%. This suggests that mutants may have dominant secretion problems or thiol reactivity issues depending on location of mutations; this interpretation could be affected by interrelationships between secretion and thiol reactivity. Third, we observe that non-cysteine pathological NOTCH3 mutations appear to have abnormalities in parameter one and parameter 2. The last finding indicates that even non-cysteine mutants alter thiol reactivity of NOTCH3. This implicates thiol reactivity as a CADASIL-associated abnormality that may link all NOTCH3 mutants, regardless of amino acid change or location.

The derivation of three LSL parameters provides a richer portrait of the biochemical abnormalities caused by CADASIL mutants. Prior work using gel shifting does not provide information on secretion efficiency and levels of protein production. As such, LSL provides significant new advantages for NOTCH3 analysis.

### LSL for other genetic disorders

We applied LSL to FBN1 to investigate the ability to discriminate pathological from benign mutants related to Marfan’s disease and Stiff Skin Syndrome. In Marfan’s, we found that the ability to distinguish pathologically from benign variants of FBN1 was incomplete. The specificity of parameters 1 to 3 for pathogenic FBN1 mutations was 100%; however, the sensitivity for discrimination of pathogenic mutations was only 44%, 41%, and 15%. It should be noted that Marfan’s disease mechanisms are thought to be related to decreased expression of FBN1; in addition, unlike CADASIL, Marfan’s disease is caused by many non-cysteine mutations. Thus, our LSL assessments of NOTCH3 and FBN1 suggest that the fundamental biochemical mechanisms that drive CADASIL and Marfan’s disease, though both are degenerative vascular diseases, are not identical.

For Stiff Skin Syndrome, though there are limited number of mutations, LSL-FBN1 provided a clear difference between mutant and wildtype FBN1 sequences. All the parameters for SSS LSL-FBN1 were abnormal, indicating that like CADASIL, protein biochemical changes in SSS may involve aberrant thiol reactivity. Overall, the utility of LSL to discriminate between pathogenic and benign variants is high for CADASIL; however, application to other disorders must be determined on a case-by-case basis in future work.

### Future studies

As a quantitative test, LSL has the potential to determine the degree of protein dysfunction. This may be useful in CADASIL, as it has recently become clear that different mutations result in higher risk of severe disease (16, 17, 18). The potential to biochemically verify this is attractive but will require careful phenotyping in large groups of patients. As a first step, we have shown that several EGF repeats can be analyzed by LSL. In addition, there appears to be baseline differences in parameter two for different regions of NOTCH3, an indication that thiol reactivity of the protein differs along the expanse of the gene product. Since the LSL assay permits quantification of the effects of mutations with expanded dynamic range compared to gel shifting, future analysis across the NOTCH3 protein using LSL may permit a refined comparison of thiol reactivity of NOTCH3 mutants across independent EGF domains that map to different clinical severity.

We find it noteworthy that cysteine alkylation increased LSL-NOTCH3 activity in all pathological mutants but not in benign variants of NOTCH3 EGF 1 to 3. Two non-cysteine mutants that have been linked to CADASIL, R75P and R61W, demonstrated significant enhancement with iodoacetamide treatment, with the former exhibiting the highest magnitude of response; this is consistent with a role of thiol reactivity in both cysteine and non-cysteine CADASIL mutations. Further, these results indicate that scaling up the LSL system may be a feasible approach to identifying cysteine reactive compounds that bind to NOTCH3 mutants, an objective that may contribute to screens for therapies. More generally, adaptation of LSL to other situations in which pathological variants are distinguished from wildtype could be useful in identification of small molecule modifiers that alter protein structure related to inherited disease.

## Experimental procedures

### DNA constructs

Expression constructs were built in the pCMV-Sport6 vector. The basic LSL construct was cloned in sequential steps that combined synthetic genes, PCR, and standard ligation. Antibody light chains were produced by either PCR or gene synthesis of rabbit variable domains of IgG light chain sequences that were originally generated against a cleavage fragment of NOTCH3 (19). The gene synthesis or PCR approach incorporated SmBiT followed by a cloning site that enabled ligation of NOTCH3 fragments into the C-terminus of the SmBiT, whose stop codon was removed. LgBiT was synthesized by PCR and cloned by ligation; a stop codon was inserted at the C-terminus of this cassette.

Point mutations in NOTCH3 were derived by PCR of templates from mutants described (7) or by nested PCR using oligos harboring specific mutations. DNA fragments were digested with appropriate restriction enzymes and cloned by ligations using T4 ligase. All clones were validated by sequencing of the inserted mutant genes.

Light chain mutation clones were generated by nested PCR onto the 83G backbone. Small insertions and mutations were created by incorporating corresponding alterations in oligonucleotides used for DNA amplification. Addition of the constant domain of the 83G light chain was accomplished by PCR.

### Cell culture

HEK293 cells (293A; Qbiogene) were propagated in DMEM with 10% fetal bovine serum in 5% carbon dioxide chambers. Transfection was performed using PolyJet as recommended by the manufacturer. Briefly, 400 ng of LSL DNA and 100 ng of iRFP expression plasmid were mixed in 25 μl serum free DMEM. DMEM (25 μl) with 1.5 μl PolyJet was mixed with diluted DNA and then added to media of cells in 24 well plates. After 6 to 12 h, the media was changed to OptiMEM. After a further 36 to 48 h, the conditioned media was analyzed for nanoLuc activity.

### LSL analysis workflow

In all experiments, we included reference groups that included the appropriate wildtype NOTCH3 sequence cloned into the LSL vector. Parameter 1 (secreted nanoLuc activity normalized to iRFP) was determined by mixing 25 μl conditioned media with 6.25 μl reaction mixture in a white 96 well plate. Luciferase activity was determined on a plate reading luminometer (BioTek Synergy LX multi-mode reader). In noted experiments, the Luc/iRFP ratio was normalized to wildtype that was set to 1.0. For some experiments, we determined Parameters 2 and 3 as follows. For Parameter 2, 2 μl of TCEP (31.25 mM) was added to the reaction mixture and repeated measurements of luciferase activity were determined over 30 min. A reaction curve was fitted to the equation: Y=Y0+(Plateau-Y0)∗(1-exp(-K∗x)) and the plateau value was determined. This was considered the Max level, which the initial value before adding TCEP was deemed the Min level. The Min/Max ratio was determined, with the working model that it was the percentage of protein in normal conformation relative to total protein. In noted experiments, the Min/Max was normalized to wildtype that was set to 1.0. For Parameter 3, cell lysates were prepared in 40 μl Lumit Immunoassay Lysis Detection Kit buffer and 0.5 to 5 μl of lysate was assayed for nanoLuc activity. The assay was then treated with TCEP and the plateau level of activity was determined as in Parameter 2. The ratio of TCEP treated secreted activity to lysate activity was considered the secretion index for the LSL protein. In noted experiments, the Secretion Index was normalized to wildtype that was set to 1.0.

### Data presentation and statistics

All data are displayed as means with standard deviations, and each point within scatterplots represents a single independent experiment. We determined normality using the Shapiro-Wilk test. For two group comparisons, we used either the *T* test or the Mann-Whitney *U* test, as appropriate. For more than two group comparisons, significant differences were determined for parametric data using one-way ANOVA with Dunn’s multiple comparisons test. Non-parametric data were evaluated using the Kruskal-Walli’s test. All analysis was performed using GraphPad Prism 8.0.0 (224). *p* value < 0.05 was considered statistically significant.

## Data availability

Experimental data and NOTCH3 cDNA sequences generated for this study are available upon reasonable request.

## Conflicts of interest

The authors declare that they have no conflicts of interest with the contents of this article.
